# Ambulante Pflege in den ersten beiden Wellen der COVID-19-Pandemie: Herausforderungen für Personal und Pflegebedürftige

**DOI:** 10.1007/s00103-023-03658-8

**Published:** 2023-02-08

**Authors:** Benedikt Preuß, Annika Schmidt, Kathrin Seibert, Viktoria Hoel, Dominik Domhoff, Franziska Heinze, Henrik Wiegelmann, Heinz Rothgang, Karin Wolf-Ostermann

**Affiliations:** 1 SOCIUM Forschungszentrum Ungleichheit und Sozialpolitik, Abteilung Gesundheit, Pflege und Alterssicherung, Universität Bremen, Mary-Somerville-Straße 1, 28359 Bremen, Deutschland; 2 Institut für Public Health und Pflegewissenschaft, Abteilung Pflegewissenschaftliche Versorgungsforschung, Universität Bremen, Bremen, Deutschland; 3 Leibniz Wissenschaftscampus Digital Public Health Bremen, Bremen, Deutschland; 4 Wissenschaftsschwerpunkt Gesundheitswissenschaften, Universität Bremen, Bremen, Deutschland

**Keywords:** COVID-19, Langzeitpflege, Ambulante Pflegedienste, Pflegebedürftige, Querschnittstudie, COVID-19, Long-term care, Home-care services, People in need of long-term care, Cross-sectional study

## Abstract

**Einleitung:**

In der COVID-19-Pandemie zählen Pflegebedürftige zu den besonders vulnerablen Bevölkerungsgruppen. Ambulante Pflegedienste befanden sich gerade zu Beginn der Pandemie in einer Ausnahmesituation. In dieser Arbeit sollen die Situation und die Probleme der Pflegedienste und der versorgten Pflegebedürftigen in den ersten beiden Wellen der Pandemie in Deutschland untersucht werden.

**Methoden:**

Während der ersten beiden COVID-19-Wellen wurden zwei Querschnittstudien durchgeführt (erste Befragung: 28.04.–12.05.2020, zweite Befragung: 12.01.–07.02.2021). Insgesamt wurden Daten aus *N* = 1029 ambulanten Pflegediensten in die Analyse einbezogen. Die Analyse erfolgte anhand deskriptiver Maßzahlen.

**Ergebnisse:**

Die Klient:innen von Pflegediensten waren in den ersten beiden Wellen der Pandemie stark belastet. Dies zeigt sich einerseits an einem erhöhten Erkrankungsrisiko und einer erhöhten Mortalität und andererseits am Wegfall verschiedener Versorgungs- und Unterstützungsangebote. Letzteres hat z. B. auch negative Auswirkungen auf die psychosoziale Verfassung der Pflegebedürftigen. Die Pflegedienste waren von hohen Personalausfällen und zusätzlicher Arbeit durch Schutzmaßnahmen betroffen.

**Diskussion:**

Die COVID-19-Pandemie führte zu großen Belastungen von Pflegebedürftigen und ambulanten Pflegediensten und zu einer Reduzierung der Versorgungsangebote. Die Verschlechterung der Versorgung traf auf eine bereits angespannte Situation. Es zeigt sich, dass die Versorgung Pflegebedürftiger durch ambulante Pflegedienste nicht krisensicher gestaltet ist und dass zusätzliche Herausforderungen wie die einer Pandemie dramatische Folgen haben können. Zukünftig sollte es verlässliche Strukturen und schnell verfügbare Notfallpläne mit konkreten Handlungsanweisungen geben.

## Abstract (en)

**Introduction:**

During the COVID-19 pandemic, people in need of long-term care were among the most vulnerable population groups. Home-care services were under exceptional strain, especially at the beginning of the pandemic. The aim of this study is to examine the situation and problems of care services and the people in need of care during the first two waves of the pandemic in Germany.

**Methods:**

Two cross-sectional studies were conducted during the first two COVID-19 waves (first survey 28 April to 12 May 2020, second survey 12 January to 7 February 2021). In total, data from *N* = 1029 outpatient care services were included in the analysis. Descriptive measures were used for the analysis.

**Results:**

The clients of home-care services were severely burdened in the first two waves of the pandemic. This can be seen on the one hand in an increased risk of illness and increased mortality, and on the other in the loss of various care and support services. The latter also has negative effects on the psychosocial condition of those in need of care, for example. Care services were affected by high staff absenteeism and additional work due to protective measures.

**Discussion:**

The COVID-19 pandemic led to immense burdens for people in need of care and home-care services and to a reduction in care services. The deterioration of care provision met with an already tense situation. It has become clear that the provision of care for those in need of care by outpatient care services is not crisis-proof, and that additional challenges such as a pandemic can have dramatic consequences. For the future, reliable structures and readily available emergency plans should be established with concrete instructions for action.

## Einleitung

Die besondere Vulnerabilität pflegebedürftiger Menschen in der COVID-19-Pandemie wurde mit Beginn der pandemischen Situation schnell deutlich [1]. Sie liegt in deren höherem Alter und der häufig vorhandenen Multimorbidität begründet, aber auch in der höheren Infektionsgefahr durch den zwangsläufig engen körperlichen Kontakt mit Pflegenden und anderen Pflegebedürftigen. Eine besondere Gefahr bestand dabei zu Beginn der COVID-19-Pandemie, in der es kaum Möglichkeiten zur Behandlung von COVID-19 und noch keine Schutzimpfungen gab. Die Situation in der ambulanten Pflege unterscheidet sich dabei von der vollstationären Versorgung: Einerseits findet die Versorgung in den meisten Fällen in einem weniger stark institutionalisierten Rahmen und somit mit weniger engem Kontakt statt, andererseits kann bei Wegfall von Leistungen die Gefahr der Vereinsamung wachsen und sich aufgrund der weniger engmaschigen Versorgung die Stabilität der Versorgung verringern, ohne dass dieser Stabilitätsverlust immer sofort bemerkt wird. Um die Situation der Pflegedienste und deren Klient:innen genauer zu erfassen, wurden im Rahmen der Studie „Zur Situation der Langzeitpflege in Deutschland während der Corona-Pandemie“ der Universität Bremen in den ersten beiden Wellen der Pandemie Befragungen von Pflegediensten in Deutschland durchgeführt.

Im Folgenden wird zur besseren zeitlichen Einordnung zunächst die pandemische Situation in Deutschland zu den beiden Befragungszeitpunkten beschrieben.

### Situation in der ersten COVID-19-Welle

Der Beginn der ersten COVID-19-Welle in Deutschland kann auf das Ende der 10. Kalenderwoche 2020 datiert werden, als die kumulierte Inzidenz erstmals den Wert von 1000 überschritten hat. Erste Lockerungen des am 23.03.2020 begonnenen bundesweiten Lockdowns in der 20. Kalenderwoche markieren das Ende der ersten COVID-19-Welle [2]. Bis zum Ende der ersten COVID-19-Welle hatten sich 174.355 Personen nachweislich mit SARS-CoV‑2 infiziert und es gab insgesamt 7914 Todesfälle im Zusammenhang mit einer COVID-19-Infektion [3]. Insgesamt lag die Anzahl der kumulierten Fälle pro 100.000 Einwohner:innen in Deutschland bei 210, wobei deutliche Unterschiede zwischen den Bundesländern vorlagen. Die Spannweite reichte von 46 in Mecklenburg-Vorpommern bis 347 in Bayern [3]. Zum Schutz der Bevölkerung wurden vor allem Einschränkungen des öffentlichen Lebens wie Absagen von Veranstaltungen, Schließungen von Kindertagesstätten und Schulen sowie weitere umfassende Kontaktbeschränkungen und Schließungen umgesetzt [4]. Schutzimpfungen und Antigentests standen in Deutschland noch nicht zur Verfügung, Schutzmaterialien, wie medizinischer Mund-Nasen-Schutz und FFP2-Masken, waren nur in geringem Maße vorrätig [5].

### Situation in der zweiten COVID-19-Welle

Der Beginn der zweiten COVID-19-Welle in Deutschland wurde auf die 40. Kalenderwoche 2020 datiert, ihr Ende auf die 8. Kalenderwoche 2021 [2]. Bis zum Ende dieser Woche waren in Deutschland 2,4 Mio. Personen nachweislich mit SARS-CoV‑2 infiziert und 70.045 mit einer solchen Infektion verstorben. Die Zahl der kumulierten Fälle in Deutschland lag bis zu diesem Datum bei 2937 pro 100.000 Einwohner:innen. Die Unterschiede zwischen den Bundesländern fielen deutlich geringer als noch in der ersten COVID-19-Welle aus: Während der Wert in Schleswig-Holstein mit 1462 pro 100.000 Einwohner:innen am niedrigsten lag, wurde der höchste Wert in Thüringen mit 3576 pro 100.000 Einwohner:innen verzeichnet [6]. Im Laufe des Jahres 2020 sind erste besorgniserregende Virusvarianten (Variants of Concern – VOC) des SARS-CoV‑2 (Alpha‑, Beta‑, Gamma- und Delta-Variante) entdeckt und benannt worden.^1^

Im Zuge einer verbesserten Datenlage zu Gefahren durch COVID-19 und Verbreitungswegen von SARS-CoV‑2 wurden Gesetze und Empfehlungen zum persönlichen Schutz sowie zum Schutz von Pflegebedürftigen in Einrichtungen kontinuierlich angepasst.^2^ Zur Früherkennung von infizierten Personen standen ab dem 15.10.2020 Antigentests für Mitarbeitende, Besucher:innen und Bewohner:innen ambulanter bzw. stationärer Pflegeeinrichtungen zur Verfügung. Außerdem wurde im Dezember 2020 in Deutschland der erste Impfstoff gegen SARS-CoV‑2 zugelassen, der den Beginn der Impfkampagne am 27.12.2020 ermöglichte. Personen über 80 Jahre und Bewohner:innen von Pflegeeinrichtungen wurden aufgrund ihrer Vulnerabilität priorisiert geimpft.^3^

### Zielsetzung und Fragestellungen

In der vorliegenden Arbeit soll die Situation ambulanter Pflegedienste (PD) und der von ihnen versorgten Pflegebedürftigen während der ersten beiden COVID-19-Wellen untersucht werden. Die leitenden Fragestellungen der Analyse lauten: Welche COVID-19-Prävalenz und COVID-19-assoziierte Mortalität unter ihren Klient:innen berichten PD? Welche Auswirkungen der COVID-19-Pandemie auf Versorgungssituation und Ressourcen zeigen sich in den PD? Wie gehen PD mit Schutzmaßnahmen gegen das SARS-CoV‑2 um? Welche Unterschiede und Trends zeigen sich dabei im Zeitverlauf der COVID-19-Pandemie? Ein Vergleich der beiden Zeitpunkte sowie der Situation vor allem in Hinblick auf Ressourcen und Veränderungen in den Arbeitsprozessen der ambulanten PD ermöglicht die Identifikation langfristiger und fortdauernder Probleme in diesem Setting während der Pandemie.

## Methodik

Datengrundlage für den vorliegenden Artikel sind zwei deutschlandweit durchgeführte Querschnittstudien, die vom 28.04. bis zum 12.05.2020 (1. Befragung) und vom 12.01. bis zum 07.02.2021 (2. Befragung) durch die Universität Bremen erfolgten. Die Online-Befragungen von ambulanten PD enthielten sowohl offene als auch geschlossene Fragen und erfassten neben Strukturmerkmalen der PD die Auswirkungen der Pandemie und den Umgang mit der Situation. Für die 2. Befragung erfolgte aufgrund von Erfahrungen aus der 1. Befragung und von äußeren Veränderungen (etwa der Einsatz von Antigentests) eine Anpassung des Fragebogens. Informationsschreiben mit Erläuterungen zur Studie und Informationen zum Datenschutz sowie Befragungslinks wurden an 9547 (1. Befragung) bzw. 9808 ambulante PD (2. Befragung) verschickt. Zudem wurde über Interessenvertretungen und Anbieterverbände für die Teilnahme an der Befragung geworben. Die Befragungen wurden mit der Software EFS-Survey, Version Fall 2019 [7] durchgeführt. In Abstimmung mit der Datenschutzbeauftragten der Universität Bremen wurde ein Datenschutzkonzept erstellt. Die Teilnehmenden wurden vorab über dieses Datenschutzkonzept informiert und mussten vor Beginn der Befragung ihre Einwilligung zur Erhebung und Nutzung der Daten erteilen. Eine Woche nach der jeweils ersten Einladung erfolgte eine Teilnahmeerinnerung per E‑Mail. Ergebnisse der 1. Befragung 2020 zu Erhebungen in ambulanten PD, aber auch stationären Einrichtungen wurden bereits an anderer Stelle publiziert [5, 8–10].

Der vorliegende Beitrag berichtet Ergebnisse der Online-Befragungen der ambulanten PD beider Befragungen. Nach Ausschluss von PD, die ausschließlich Angaben zum PD und keine Angaben zu inhaltlichen Fragen gemacht hatten, verblieben *N* = 701 PD der 1. Befragung und *N* = 442 PD der 2. Befragung, die in die Auswertung eingingen. *N* = 114 Teilnehmer:innen der 2. Befragung gaben an, auch an der 1. Befragung teilgenommen zu haben. Insgesamt gingen somit Angaben von 1029 verschiedenen PD in die Auswertung ein. Die erhobenen Antworten wurden auf Plausibilität geprüft, Werte außerhalb des gültigen Wertebereichs wurden ebenso wie fehlende Werte für die Auswertung der jeweiligen Items ausgeschlossen. Entsprechend wird im Ergebnisteil jeweils das für die Prozentberechnung verwendete *N* der berücksichtigten, gültigen Fälle angegeben. Absolute Antworthäufigkeiten sind mit *n* gekennzeichnet. Die deskriptive Analyse unter Verwendung von Lage- und Streuungsparametern wurde mit der Software SAS Version 9.4 durchgeführt.

## Ergebnisse

### Stichprobenbeschreibung

Die Rücklaufquote liegt in der 1. Befragung bei 7,3 % (*N* = 9547) und in der 2. Befragung bei 4,5 % (*N* = 9808). In beiden Befragungen sind PD aus allen Bundesländern vertreten. In Relation zu der in der Pflegestatistik 2019 aufgeführten Zahl der PD [11] lag der Anteil der teilnehmenden PD zwischen 0,6 % (Thüringen, *n* = 3) und 6,0 % (Bayern, *n* = 124). Die einbezogenen PD versorgten 82.949 (1. Befragung) bzw. 51.670 (2. Befragung) Klient:innen und setzten dabei 26.261 (1. Befragung) bzw. 15.320 (2. Befragung) Mitarbeitende ein. Die durchschnittliche Anzahl von Klient:innen liegt somit bei 118,3 (Median = 100; Q1^4^ = 58; Q3^5^ = 159) in der 1. Befragung bzw. 116,9 (Median = 95; Q1^6^ = 55; Q3^7^ = 156) in der 2. Befragung. Die Stichproben beider Befragungen sind in Bezug auf die Gesamtzahl der Mitarbeitenden und die Anzahl der Pflegekräfte sehr ähnlich: Im Vergleich zur amtlichen Statistik fällt auf, dass die PD in den vorliegenden Stichproben gemessen an der Anzahl der Mitarbeitenden deutlich größer sind. In Bezug auf die Trägerschaft fällt eine geringfügige Überrepräsentation der öffentlichen PD in der 1. Befragung sowie der privaten PD in der 2. Befragung auf (Tab. 1).

**Table 1:** 

	1. Befragung			2. Befragung			Pflegestatistik 2019
–	Arithmetisches Mittel	Median	Q1–Q3	Arithmetisches Mittel	Median	Q1–Q3	–
*Mitarbeitende gesamt*							
Personenzahl (*N* = 613/*N* = 387)	37,5	25	15–45	34,7	22	14–40	28,7
Vollzeitäquivalente (*N* = 579/*N* = 374)	19,8	14	7–24	18,1	13	7–21	19,6
*Pflegekräfte*							
Personenzahl (*N* = 595/*N* = 381)	25,8	17	10–30	24,3	15	9–26	20,7
Vollzeitäquivalente (*N* = 543/*N* = 354)	15,5	10	6–20	13,5	9	5–17	14,3
*Trägerschaft* *N* = 674 (1. Befragung) bzw. *N* = 428 (2. Befragung)							
–	Anteil (%)		Anzahl	Anteil (%)		Anzahl	Anteil (%)
Öffentlich	5,0		34	3,3		14	1,3
Freigemeinnützig	33,8		228	26,2		112	32,1
Privat	61,1		412	70,6		302	66,5

*Q1* erster Quartilswert; *Q3* dritter Quartilswert

### Verbreitung von COVID-19 unter Pflegebedürftigen und Mitarbeitenden

Während in der 1. Befragung nur jeder sechste PD von Infektionen der Klient:innen und weniger als jeder zehnte PD von Todesfällen unter Klient:innen bzw. Infektionen der Mitarbeitenden betroffen war, stiegen diese Zahlen mit der 2. Befragung stark an. Zu diesem Zeitpunkt waren in zwei Dritteln der PD Infektionen unter Klient:innen sowie in einem Drittel der PD unter Mitarbeitenden aufgetreten. In mehr als der Hälfte der PD sind zudem Todesfälle unter Klient:innen aufgetreten (Tab. 2).

**Table 2:** 

PD mit …		Infizierten Klient:innen (*N* = 627; *N* = 437)		Verstorbenen Klient:innen (*N* = 599; *N* = 442)		Infizierten Mitarbeitenden (*N* = 627; *N* = 437)	
		Anteil der betroffenen PD	Fälle	Anteil der betroffenen PD	Fälle	Anteil der betroffenen PD	Fälle
*1. Befragung*							
Stichprobe		16,7 %	250	8,7 %	81	9,0 %	116
Hochrechnung^a^	Untere Grenze 95 %-KI	–	2592	–	752	–	1405
	Punktschätzer	–	2958	–	961	–	1717
	Obere Grenze 95 %-KI	–	3325	–	1170	–	2030
*2. Befragung*							
Stichprobe		66,4 %	1348	29,4 %	278	56,5 %	800
Hochrechnung	Untere Grenze 95 %-KI	–	24.285	–	4664	–	20.532
	Punktschätzer	–	25.636	–	5284	–	22.018
	Obere Grenze 95 %-KI	–	26.986	–	5903	–	23.503

*KI* Konfidenzintervall

^a^ Die Hochrechnung der ersten Befragung wurde im Vergleich zur ersten Publikation [5] mithilfe der Pflegestatistik 2019 [11] aktualisiert. Aus diesem Grund ergeben sich diesbezüglich leichte Abweichungen.

Werden die in den Stichproben bezifferten Fälle bzw. Todesfälle anhand der Gesamtzahl von Klient:innen bzw. Mitarbeitenden ambulanter PD in Deutschland hochgerechnet und dann in Relation zur Gesamtzahl der Infizierten bzw. mit COVID-19 Verstorbenen gemäß den Angaben des Robert Koch-Instituts (RKI) gesetzt, zeigt sich, dass der Anteil der infizierten Klient:innen und Mitarbeitenden jeweils rund 1–2 % der insgesamt bis zu diesen Zeitpunkten^8^ in der Pandemie identifizierten Fälle ausmachte. Da der Anteil der in ambulanten PD beschäftigten Personen nur 0,5 % der Bevölkerung^9^ ausmacht, war das Infektionsrisiko für diese Personengruppe somit in der 1. Befragung um den Faktor 1,6, in der 2. Befragung um den Faktor 2,5 höher. Das Risiko für Klient:innen, die einen Anteil von rund 1,2 % der Bevölkerung ausmachen, lag zu den beiden Zeitpunkten um den Faktor 1,5 bzw. 1,2 höher. In Bezug auf die Todesfälle unter Klient:innen zeigt sich, dass in der 1. Befragung 14 % aller Todesfälle in Deutschland und in der 2. Befragung 11 % aller Todesfälle auf Klient:innen von PD zurückzuführen sind.

### Die Versorgungssituation und Auswirkungen auf Pflegebedürftige

Tab. 3 stellt die berichteten Veränderungen bei der Inanspruchnahme von Versorgungsleistungen dar. Bei der 1. Befragung zielte die Frage zum Inanspruchnahmeverhalten auf eine Beurteilung im Vergleich zur Zeit vor Beginn der Pandemie ab, in der 2. Befragung sollte eine entsprechende Einschätzung für die letzten sechs Monate abgegeben werden. Bei beiden Befragungen wird deutlich, dass sich die Inanspruchnahme fast aller Leistungsarten in einem Großteil der PD verändert hat. Während sich die Inanspruchnahme in der 1. Befragung (mit Ausnahme der Behandlungspflege) mehrheitlich verändert hat und dabei in bis zu 65 % der PD rückläufig war, ist in der 2. Befragung sowohl der Anteil der PD mit Veränderungen als auch der Anteil mit einem Rückgang deutlich geringer (Tab. 3).

**Table 3:** 

		1. Befragung		2. Befragung	
Leistungsart	Veränderung	Anteil der PD (%)	Anzahl der PD	Anteil der PD (%)	Anzahl der PD
Behandlungspflege nach SGB V (*N* = 605/*N* = 420)	Abnahme	35,0	212	16,7	70
	Keine Veränderung	52,4	317	49,8	209
	Zunahme	12,6	76	33,6	141
Grundpflege nach SGB XI (*N* = 628/*N* = 423)	Abnahme	46,5	292	27,9	118
	Keine Veränderung	40,3	253	40,0	169
	Zunahme	13,2	83	32,2	136
Betreuungsmaßnahmen (*N* = 604/*N* = 406)	Abnahme	65,4	395	35,5	144
	Keine Veränderung	22,2	134	38,9	158
	Zunahme	12,4	75	25,6	104
Hauswirtschaftliche Versorgung (*N* = 600/*N* = 404)	Abnahme	64,2	385	25,0	101
	Keine Veränderung	24,5	147	31,2	126
	Zunahme	11,3	68	43,8	177
Serviceleistungen (*N* = 533/*N* = 350)	Abnahme	44,1	235	22,3	78
	Keine Veränderung	36,8	196	47,4	166
	Zunahme	19,1	102	30,3	106
Beratung für Pflege und Betreuung (*N* = 617/*N* = 424)	Abnahme	63,5	392	29,5	125
	Keine Veränderung	23,2	143	35,8	152
	Zunahme	13,3	82	34,7	147

*SGB V* Fünftes Buch Sozialgesetzbuch, *SGB XI* Elftes Buch Sozialgesetzbuch

Die PD wurden zudem gebeten, die Versorgungssituation der Personen, bei denen die Inanspruchnahme zurückgegangen ist, mithilfe einer 3‑stufigen Skala einzuschätzen (Versorgung sichergestellt – Versorgung gefährdet/instabil – Versorgung nicht sichergestellt). Während die Versorgung der Personen mit rückläufiger Inanspruchnahme in der 1. Befragung noch bei über 50 % dieser Personen als sichergestellt eingeschätzt wurde, sank der Anteil in der 2. Befragung auf weniger als 40 %. Zugleich wuchs der Anteil der Personen, deren Versorgung als gefährdet/instabil eingeschätzt wurde, von 37,5 % in der 1. Befragung auf 45,4 % in der 2. Befragung sowie der Anteil der Personen, deren Versorgung als nicht sichergestellt eingeschätzt wurde, von 8,3 % auf 15,4 % (*N* = 552/*N* = 377).

Die PD wurden befragt, wie groß sie retrospektiv den Personalausfall aufgrund der COVID-19-Pandemie (inkl. etwaiger Quarantänemaßnahmen) im April 2020 (1. Befragung) bzw. im Dezember 2020 (2. Befragung) einschätzen. Im April 2020 wurde in 57,3 % der PD (*N* = 609) von Personalausfällen berichtet, im Dezember 2020 waren fast drei Viertel der PD davon betroffen (73,1 %; *N* = 427). Im arithmetischen Mittel (gebildet mit Hilfe der Klassenmitten) lag der Personalausfall in der 1. Befragung bei 7,0 % und in der 2. Befragung bei 8,9 %. Insgesamt kam es in der 1. Befragung in jedem zehnten PD zu Personalausfällen von 10 % oder mehr. In der 2. Befragung war jeder fünfte PD von einem solchen Personalausfall betroffen. Der Anteil der PD, in denen es seit Beginn der Pandemie (1. Befragung) bzw. in den sechs Monaten vor der 2. Befragung pandemiebedingt zu einem Aufnahmestopp für neue Klient:innen gekommen ist, lag in der 1. Befragung bei 36,6 % (*N* = 618) und in der 2. Befragung bei 40,4 % (*N* = 423). In 58,4 % (*N* = 296) der PD wurden in der 2. Befragung zudem soziale Angebote für Menschen mit Demenz eingestellt.

In der 1. Befragung berichteten rund 40 % der PD, dass in mindestens einem der von ihnen versorgten Haushalte bezahlte Hilfs‑/Betreuungskräfte aus dem Ausland (Live-Ins) leben. Ein Drittel dieser PD berichtet von einem Wegfall dieser Hilfskräfte im Zuge der Pandemie. Zudem waren teilstationäre Betreuungsangebote durch Aufnahmestopps und Schließungen während der 1. Befragung stark reduziert. Von Schließungen teilstationärer Einrichtungen wurde in der 2. Befragung nur in Einzelfällen (2,5 %; *N* = 80) und von Aufnahmestopps in Bezug auf die letzten 6 Monate von 59,2 % (*N* = 76) der teilstationären Einrichtungen berichtet.

**Figure 1:**
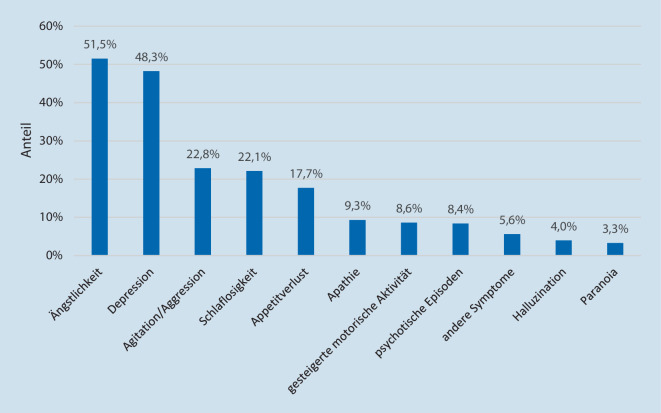

In der 2. Befragung wurde nach dem Anstieg neuropsychiatrischer Symptome bei Menschen mit Demenz während der letzten sechs Monate gefragt. Die Ergebnisse dazu werden in Abb. 1 dargestellt. Es wird deutlich, dass von rund der Hälfte der PD ein Anstieg von Ängstlichkeit und Depressionen innerhalb dieser Personengruppe beschrieben wird. Zudem wird von fast jedem vierten PD eine Steigerung der Symptome Agitation/Aggression und Schlaflosigkeit sowie von jedem sechsten Dienst eine Zunahme von Appetitverlust beobachtet. Ein Anstieg weiterer neuropsychiatrischer Symptome wird nur von wenigen PD beobachtet. Von 25,5 % der PD (*N* = 290) wurde bei Menschen mit Demenz zudem ein Anstieg der medikamentösen Behandlung bemerkt.

### Maßnahmen zum Infektionsschutz

Im Rahmen der 1. Befragung wurde deutlich, dass ein Großteil der PD nicht auf eine Pandemie vorbereitet war. Als Reaktion auf die pandemische Situation wurden in zwei Dritteln der PD Krisenteams gebildet. Zudem wurden in rund drei Vierteln der PD Schulungen zu Hygienemaßnahmen sowohl für Mitarbeitende als auch für Klient:innen und deren Angehörige ebenso wie Abwesenheitsregelungen für Mitarbeitende mit typischen Symptomen umgesetzt (s. auch [5]).

Eine der wesentlichen Schutzmaßnahmen, die während der zweiten COVID-19-Welle zusätzlich Einzug hielt, war die Verwendung von Antigentests, um kurzfristig eine Infektion mit SARS-CoV‑2 mit einer gewissen Wahrscheinlichkeit auszuschließen. Die meisten PD geben an, regelmäßig symptomunabhängige Testungen bei Mitarbeitenden durchzuführen, in 18,1 % der PD täglich, in 71,0 % der PD wöchentlich. In *n* = 34 PD (7,7 %) wurden Mitarbeitende nur im Fall von Ausbrüchen innerhalb des PD bzw. bei Kontakt zu Fällen außerhalb des PD durchgeführt, in *n* = 14 PD (3,2 %) erfolgte gar keine symptomunabhängige Testung. In rund einem Drittel der PD erfolgten anlassbezogene Testungen bei Kontakt zu Fällen bzw. bei Neueinstellung zusätzlich zu den regelmäßigen Testungen (*N* = 436). Die Durchführung der Antigentests wurde in mehr als neun von zehn PD von Pflegefachkräften der PD, in Einzelfällen durch Pflegehilfskräfte der PD und in 5,8 % der PD von externen Dienstleistern übernommen (*N* = 432). Der zusätzliche Zeitaufwand für die Durchführung von Antigentests in den PD belief sich täglich auf durchschnittlich 2,3 h. Von den PD wurden umfangreiche Auswirkungen der Tests berichtet: Einerseits berichteten mehr als drei Viertel der PD (80,1 %) von einem höheren Sicherheitsgefühl und 43,9 % von einer im Vergleich schnelleren Umsetzung von Infektionsschutzmaßnahmen bei positiven Tests. Andererseits wurde in 22,2 % der PD von Unterbrechungen der direkten Versorgung, in 43,2 % von einem erhöhten Schulungs‑/Informationsbedarf und in 71,0 % der PD von Unterbrechungen der Arbeitsorganisation berichtet (jeweils *N* = 428). In den meisten PD (69,2 %; *N* = 442) konnten die Testungen ohne Schwierigkeiten durchgeführt werden. In rund 10 % der PD wurde von Schwierigkeiten bei der Umsetzung von Antigentests durch Personalmangel, Materialmangel und durch Verweigerung der zu testenden Personen berichtet (*N* = 424).

**Figure 2:**
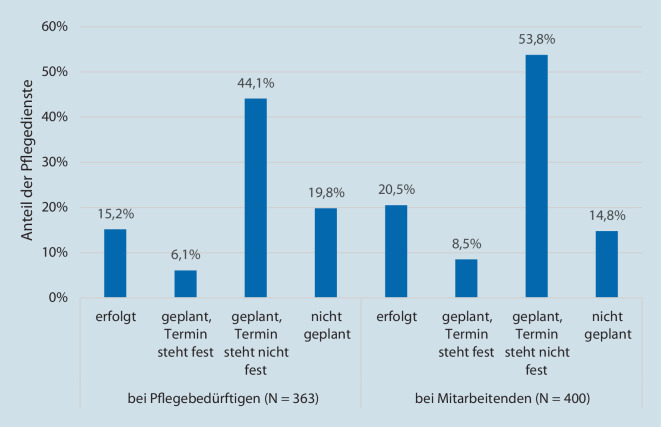

Eine weitere bedeutende Schutzmaßnahme ist die Impfung gegen COVID-19. Auch wenn die 2. Befragung nur wenige Wochen nach Beginn der Impfkampagne in Deutschland durchgeführt wurde, wurde in dieser Erhebung bereits der Impfstand unter Pflegebedürftigen und Mitarbeitenden der PD abgefragt. In den meisten PD waren Impfungen zum Zeitpunkt der Befragung geplant, aber es standen noch keine Termine dafür fest. In jedem fünften PD waren noch keine Impfungen bei Pflegebedürftigen geplant. Die Planung von Impfungen für Mitarbeitende hatte in jeder siebten PD noch nicht stattgefunden. In 20,5 % der PD waren bereits Impfungen bei Mitarbeitenden und in 15,2 % der PD Impfungen bei Pflegebedürftigen erfolgt (Abb. 2). Durchschnittlich lag die Impfquote in diesen PD unter den Mitarbeitenden bei 35,1 % (*N* = 71) und unter den Pflegebedürftigen bei 17,5 % (*N* = 51). In 69,5 % der PD wurde die Impfung von einigen Pflegebedürftigen (*N* = 361) und in 83,1 % der PD von einigen Mitarbeitenden (*N* = 419) abgelehnt.

## Diskussion

### Zentrale Ergebnisse

In beiden Befragungen zeigt sich vor allem in Hinblick auf die Mortalität im Zusammenhang mit COVID-19, dass Pflegebedürftige, die von ambulanten PD versorgt werden, stark betroffen sind. Zudem fällt in der 1. Befragung auf, dass es im Vergleich zum Status quo ante zu deutlichen Rückgängen in der Inanspruchnahme von Leistungen kam. In der 2. Befragung war in Bezug auf die Inanspruchnahme von Leistungen während der sechs Monate vor der 2. Befragung vermehrt ein Anstieg zu beobachten, in einigen PD jedoch auch weiterhin Abnahmen. Die Versorgungssituation von Pflegebedürftigen, die weniger Leistungen in Anspruch genommen haben, verschlechterte sich von der 1. zur 2. Befragung deutlich. Zudem zeigt sich über beide Befragungen ein Wegfall von zusätzlichen Versorgungsangeboten. Weiterhin sind in der 2. Befragung eine weitere Verschlechterung der Personalsituation im Vergleich zur 1. Befragung erkennbar sowie ein vermehrtes Auftreten von Aufnahmestopps. Bei den Pflegebedürftigen mit einer dementiellen Erkrankung werden negative Auswirkungen sowohl in Bezug auf die Symptomatik als auch in Form von zusätzlich verabreichter Medikation deutlich.

Insgesamt können bei den ambulant versorgten Pflegebedürftigen erhebliche Auswirkungen der Pandemie bereits zu Beginn, aber auch in deren Verlauf festgestellt werden. In Bezug auf den Umgang mit der Pandemie hat sich in der 1. Befragung gezeigt, dass die PD zunächst nicht gut vorbereitet waren, sie dann aber die Schutzmaßnahmen gut umsetzen konnten. In Bezug auf Schutzmaßnahmen zeigt sich unter einigen Mitarbeitenden und Klient:innen zu Beginn der Impfkampagne eine eher zurückhaltende Einstellung gegenüber Schutzimpfungen, zudem wird deutlich, dass es in manchen Fällen zu Problemen bei der Durchführung von Antigentests gekommen ist.

### Interpretation und Einordnung in den Stand der Forschung

SARS-CoV-2-Infektionen und Todesfälle mit COVID-19 stiegen in der Allgemeinbevölkerung zwischen der ersten und zweiten COVID-19-Welle stark an, was auch der Anstieg von Fällen und Todesfällen in den PD widerspiegelt. Abschätzungen auf Grundlage der Meldedaten [14] sowie Hochrechnungen der Ergebnisse der 1. Befragung [8] weisen darauf hin, dass bis Herbst 2020^10^ die mit COVID-19 verstorbenen Bewohner:innen von Pflegeheimen mehr als 50 % aller entsprechenden Todesfälle in Deutschland ausmachten. Die Auswertung von Kassendaten zeigt für die beiden Befragungszeiträume diesbezüglich noch höhere Anteilswerte [15]. Die hier dargestellten Hochrechnungen zeigen, dass außerdem mehr als 10 % der gesamten Todesfälle auf Klient:innen von ambulanten PD entfallen.

Die in der 2. Befragung erfasste geringe Impfquote ist darauf zurückzuführen, dass die Impfkampagne nur wenige Wochen vor der Erhebung begonnen hat, die Daten des RKI zeigen mittlerweile eine hohe Akzeptanz der Schutzimpfung in der Bevölkerung ab 60 Jahren [16], die implizit auch ambulant pflegerisch versorgte Personen umfasst. Ebenso zeigt sich eine hohe Impfquote unter Mitarbeitenden und Bewohner:innen von stationären Pflegeeinrichtungen [17]. Entsprechende explizite Daten für ambulante PD wurden noch nicht vorgelegt. Es zeigt sich außerdem ein deutlich positiver Effekt der Impfungen in Bezug auf Mortalität, intensivpflichtige Behandlungen und Hospitalisierungen in der Allgemeinbevölkerung [16] und unter Pflegebedürftigen [15].

Wie die Ergebnisse zeigen, sind Klient:innen ambulanter PD zu Beginn der Pandemie aber auch von instabilen Versorgungssituationen bedroht gewesen, die sich im Verlauf zudem teilweise verschärften. Dies deckt sich mit anderen Berichten [18–20]. Ein Erklärungsmodell für die instabile Situation wird bei Hower et al. [21] dargestellt. In Bezug auf einzelne Leistungen kam es in der 2. Befragung zu einer Entspannung der Situation. Veränderungen einzelner Faktoren im komplexen Zusammenspiel von Herausforderungen und Belastungen im Zuge der COVID-19-Pandemie, wie bspw. eine Verbesserung bei der Beschaffung von Ausrüstung zum Infektionsschutz [22], können zur Entspannung der Situation beigetragen haben.

Dagegen wurde in der ersten COVID-19-Welle von reduzierter informeller Unterstützung sowie einer Reduktion sozialer Kontakte insgesamt [18] und einer Verschlechterung der medizinischen Versorgung [19] berichtet. Da die Durchführung der Antigentests durch das Pflegepersonal der PD erfolgte und entsprechend Zeit in Anspruch nahm, ist von einer zusätzlichen Reduktion der direkten Versorgung bzw. einem erhöhten Personalbedarf auszugehen. Dies könnte wiederum die Gefahr von Mängeln in der Versorgung der Pflegebedürftigen und von deren Vereinsamung erhöht haben.

Während die Immunisierung durch Impfungen einen positiven Einfluss auf die Mortalität und auf die Schwere der Krankheitsverläufe hatte, wurde ihr Einfluss auf eine Ansteckung, der zunächst sehr groß war, mit Verbreitung der Omikron-Variante weit geringer. Entsprechend ist für die Zeit, in der die Omikron-Variante dominant ist, in Bezug auf die Versorgungssituation kaum mit Entlastungen der Pflegebedürftigen und Pflegekräfte durch die Impfungen zu rechnen. Folglich ist ein Fortbestand und ggf. eine Verschärfung der beschriebenen negativen Einflüsse auf die psychosoziale Lage der betroffenen Pflegebedürftigen, die sich auch in anderen Studien zeigen [18–20], möglich.

### Limitationen und Stärken

Die Ergebnisse basieren auf zwei Zufallsstichproben. Trotz des jeweils geringen Rücklaufs war die Anzahl antwortender PD insgesamt hoch. In Bezug auf die Größe der an der Befragung teilnehmenden PD fällt ein Unterschied zur amtlichen Statistik [11] auf, die Vergleichbarkeit hierzu ist jedoch aufgrund fehlender Differenzierung nach SGB-V/XI/XII-Leistungen^11^ nur eingeschränkt gegeben. Selbstselektionseffekte, die vor allem auch mit dem Umgang der PD mit der COVID-19-Pandemie assoziiert sind, sind dagegen nicht auszuschließen. Wie andere Untersuchungen zeigen, waren PD auch finanziell stark belastet [22, 23]. Dies könnte zu Schließungen von PD im Zeitraum zwischen den zwei Befragungen geführt haben und somit einen Selektionseffekt zur Folge gehabt haben. Außerdem waren die Befragungen an die Leitungspersonen adressiert, die evtl. in einigen Aspekten beschränkte Kenntnisse hatten und eher ihre eigene Perspektive geschildert haben.

Durch die Berücksichtigung von Daten zu zwei Zeitpunkten der Pandemie sind ein Vergleich der Ergebnisse beider Befragungen sowie eine Darstellung des Verlaufs – zumindest für die ersten COVID-19-Wellen zwischen April 2020 und Februar 2021 – möglich. Dennoch handelt es sich um Ergebnisse aus zwei Querschnittstudien, die naturgemäß eine eingeschränkte Aussagekraft zu Verläufen haben.

### Fazit

Der Artikel stellt Ergebnisse zweier Querschnittstudien in ambulanten PD zur Situation und zu Problemen in den ersten beiden COVID-19-Wellen dar. Dabei wird deutlich, dass Pflegebedürftige, die durch ambulante PD versorgt werden, direkt durch ein hohes Mortalitätsrisiko und indirekt durch reduzierte Versorgungsangebote doppelt von der Pandemie betroffen waren. Die Verschlechterung der Versorgung traf zudem auf eine bereits angespannte Situation, da bereits vor Beginn der Pandemie potenzielle Klient:innen von PD aufgrund mangelnder Kapazitäten abgelehnt werden mussten. Außerdem zeigt sich, dass sich die Situation von der 1. zur 2. Befragung weiter verschärft hat. Durch die mittlerweile fortgeschrittene Impfkampagne konnte zwar die Mortalitätsrate gesenkt werden. Aufgrund der weiterhin hohen Viruslast in der Allgemeinbevölkerung ist davon auszugehen, dass die Versorgungslage aber weiterhin entsprechend angespannt bleibt.

Insgesamt zeigt sich, dass die ohnehin vor großen Herausforderungen stehende Versorgung Pflegebedürftiger durch ambulante PD nicht krisensicher gestaltet ist und dass zusätzliche Herausforderungen wie die einer Pandemie dramatische Folgen für die Pflegebedürftigen haben können. In Hinblick auf weitere COVID-19-Wellen, andere Pandemien oder Krisen, wie bspw. durch extreme Wetterlagen und Hitzewellen, sollte es neben verlässlichen Strukturen auch Notfallpläne geben. Letztere sollten im Krisenfall schnell verfügbar sein und verbindliche Handlungsanweisungen enthalten. Nur so kann ein adäquates Katastrophenmanagement funktionieren und die Versorgung im Krisenfall aufrechterhalten werden.

## Footnotes

1 https://www.who.int/en/activities/tracking-SARS-CoV-2-variants/ [Zugegriffen am 22.08.2022].

2 https://www.bundesgesundheitsministerium.de/coronavirus/chronik-coronavirus.html [Zugegriffen am 22.08.2022].

3 https://www.bundesgesundheitsministerium.de/coronavirus/chronik-coronavirus.html [Zugegriffen am 22.08.2022].

4 Q1 = erster Quartilswert.

5 Q3 = dritter Quartilswert.

6 Q1 = erster Quartilswert.

7 Q3 = dritter Quartilswert.

8 Als Stichtage wurden der 05.05.2020 und der 20.01.2021 gewählt, da an diesen Tagen jeweils die Hälfte der Antworten eingegangen war und sich somit die Länge der Erhebungszeiträume der Stichprobe und der RKI-Daten im Mittel gleichen. Die Anzahl der kumulierten Fälle in Deutschland insgesamt lag an diesen Tagen bei 166.220 bzw. 1.741.800, die Anzahl der kumulierten Todesfälle in Deutschland insgesamt lag bei 6831 bzw. 48.770 [12, 13].

9 https://www.destatis.de/DE/Themen/Gesellschaft-Umwelt/Bevoelkerung/Bevoelkerungsstand/Tabellen/liste-zensus-geschlecht-staatsangehoerigkeit.html [Zugegriffen am 22.08.2022].

10 Bis zum 15.11.2020 waren Abschätzungen zum Anteil der verstorbenen Bewohnerschaft bzw. Klientel auf Grundlage dieser Daten möglich. Seit dem 16.11.2020 sind Informationen zu fehlenden Angaben in den Meldedaten nicht verfügbar. Deshalb ist eine entsprechende Hochrechnung seitdem nicht mehr möglich. Seit dem 08.01.2021 werden vom Robert Koch-Institut konkrete Fallzahlen in Pflegeheimen und ambulanten PD berichtet, jedoch noch immer nur für eine Teilmenge der COVID-19-Fälle.

11 In der Pflegestatistik werden Pflegebedürftige, die ausschließlich häusliche Krankenpflege oder Haushaltshilfe nach SGB V bzw. Hilfe zur Pflege nach SGB XII oder ausschließlich Leistungen der nach Landesrecht anerkannten Angebote zur Unterstützung im Alltag (§ 45b Abs. 1 S. 3 Nr. 4 SGB XI) oder ausschließlich ambulante Entlastungsleistungen (§ 45b Abs. 1 S. 3 Nr. 3 SGB XI) beziehen, nicht berücksichtigt. Diese Differenzierung wurde in diesen Befragungen nicht berücksichtigt.
